# Voluntary Hospitals Committee

**Published:** 1921-03-26

**Authors:** 


### VOLUNTARY HOSPITALS COMMITTEE.

### Interim Report by Lord Cave's Committee.

To the Right Hon. Christopher Addison, M.D., F.R.C.S., M.P., Minister of Health.

Sir,—We were appointed by your minute of January 25 last to be a Committee "to consider the present financial position of voluntary hospitals, and to make recommendations as to any action which should be taken to assist them," and we are engaged upon the inquiry. The evidence already received has convinced us that it is desirable, in the public interest, to maintain the voluntary system of hospital management, and also that—mainly owing to the large increase during and since the war of salaries and wages, and the cost of provisions and materials—many of the hospitals are faced with a serious deficiency of income to meet the necessary expenditure; and we hope, in due course, to make recommendations as to the steps which should be taken to meet this difficulty, but we think it desirable to make this interim report upon a pressing matter.

We understand that the quinquennial valuation of Approved Societies under the National Health Insurance Acts, which is now in hand, shows a considerable surplus over the amounts required for the purposes of the Acts. Excluding Ireland, the valuation of 4,926 societies and branches, containing 2,558,123 members (out of a total of approximately 10,000 Societies and branches containing nearly 15,000,000 members), which had been completed on December 24 last, showed a total disposable surplus as at December 31, 1918, of £2,171,576; and it is anticipated that when the valuation is completed the total disposable surplus of all the Approved Societies will not be less than seven million pounds. This result is attributed to a material extent to conditions arising out of the war, such as the reduction, owing to war conditions, of sickness, disablement, and maternity claims, and the release of reserves in respect of members killed on active service.

Under the provisions of the Acts the Societies are empowered to submit to the Minister of Health for approval schemes for utilising the disposable surplus in providing additional benefits during the next five years; and the list of additional benefits authorised by the Acts and the regulations made thereunder includes, besides such matters as increase of sickness, disablement, and maternity benefits, and the provision of dental treatment, the "payment of the whole or any part of the cost of the maintenance and treatment of members in a hospital or convalescent home."

We are strongly of opinion that, in the interests both of the hospitals and of the Societies, the schemes to be approved should provide for the application of a substantial part of the disposable surplus in providing a contribution towards the cost of the maintenance of members in hospitals. It will not, we think, be denied that the work of the voluntary hospitals is of great value to the Approved Societies and their members. Of the patients treated at the hospitals, a considerable portion, about 30 per cent. of the out-patients and a larger percentage of the in-patients, are insured persons, and the effect of hospital treatment is not only to relieve these patients from suffering, but also to reduce the periods during which they are a charge upon the Insurance funds. The fact that while depleting the hospital funds, the war has had the effect of greatly increasing the surplus available to the Approved Societies, seems to provide a special reason for the consideration by the Societies of the claims of the hospitals. The power given to the Societies by Section 21 of the 1911 Act to subscribe to hospitals has been little used, doubtless owing to the anxiety of those concerned in the management of Societies that their resources shall be sufficient to meet all claims; but now that a surplus is in sight it seems probable that a different view will be taken.

The method which should be adopted in order to secure the above result requires some consideration. The contribution of an Approved Society might take the form either of a weekly payment towards the cost of the maintenance of any member of the Society who might become an in-patient in a voluntary hospital, or of a quarterly or yearly subscription for the benefit of the hospital or hospitals concerned; but in the latter case some amendment of the regulations would appear to be required. In either case an arrangement might (if so desired) be made with the hospitals for relieving the members of the Society from any obligation to make further payments in respect of their maintenance or treatment.

We have reason to believe that some of the largest Approved Societies would be glad to see these proposals generally accepted, and that the hospitals would welcome such a result.

We are aware that it is not the desire of the Ministry of Health to interfere unnecessarily with the schemes submitted by the different Societies for the utilisation of their surplus funds. But we are confident that if the proposals here made were brought to the notice of the members the general feeling would be in favour of providing this measure of assistance to the hospitals, and so of making some return for the great benefits which those institutions have so long and so generously conferred upon them.

We therefore recommend that the Ministry

(1) should make any necessary amendment in the regulation;

(2) should forthwith bring this matter to the notice of the Societies concerned, and advise them as to the amount of the contributions which might reasonably be made out of the available surplus; and

(3) should not approve any scheme for the disposal of a surplus until the suggestion has received due consideration.

We should have preferred to include these recommendations in our general report, so that their relation to other parts of the report might be seen; but we understand that the matter is urgent, as some of the Societies are now engaged in framing schemes under the Act.

We have the honour to be, Sir, your obedient servants, Cave (Chairman), Linlithgow, C. Hyde, Wm. B. Peat, Vernon Hartshorn, R. C. Norman.

L. G. Brock, Secretary.

March 9, 1921.

